# Clinical effects of a novel deep learning-based rehabilitation application on cardiopulmonary function, dynamic and static balance, gait function, and activities of daily living in adolescents with hemiplegic cerebral palsy

**DOI:** 10.1097/MD.0000000000037528

**Published:** 2024-03-08

**Authors:** Yeongsang An, Seunghwa Min, Chanhee Park

**Affiliations:** a Funrehab Co., Ltd., Daejeon, Korea; b Department of Physical Therapy, Yonsei University, Wonju, Korea.

**Keywords:** balance, cardiopulmonary function, cerebral palsy, deep learning, neurorehabilitation

## Abstract

**Background:**

Adolescents with hemiplegic cerebral palsy undergo conventional physical therapy (CPT) to improve static and dynamic balance, activities of daily living and cardiopulmonary function. To overcome this problem, we developed an innovative deep learning-based rehabilitation application (DRA) to provide a motivational and chaffed platform for such individuals. DRA evaluates the patients’ functional abilities and diagnosis an appropriate therapeutic intervention like CPT.

**Methods:**

We compared the effects of DRA and CPT on 6-minute walking test (6 MWT), Borg rating of perceived exertion scale, Berg balance scale, functional ambulation category, and modified Barthel index in adolescents with hemiplegic cerebral palsy. A convenience sample of 30 adolescents with hemiplegic cerebral palsy was randomized into either the DRA or CPT group. DRA and CPT were administered to the participants, with each session lasting 30 minutes and apportioned thrice a week for a total of 4 weeks.

**Results:**

Analysis of variance was performed and the level of significance was set at *P* < .05. The analysis indicated that DRA showed therapeutic effects on 6 MWT, Berg balance scale, and modified Barthel index compared to CPT.

**Conclusion:**

Our results provide evidence that DRA can improve cardiopulmonary function, balance, and activities of daily living more effectively than CPT in adolescents with hemiplegic cerebral palsy.

## 1. Introduction

There is strong evidence that physical therapy interventions improve patients’ activities of daily living (ADL), balance, gait, and cardiopulmonary function. When paired with traditional physical therapy, deep learning-based rehabilitation appears to have promising outcomes for cardiopulmonary function, balance, gait, and ADLs.^[1–5]^ Furthermore, the labor-intensive nature of neurodevelopmental treatment-based conventional physical therapy (CPT), the physical strain it places on therapists, and the insufficient repetition needed to produce enough neuroplasticity poses practical challenges.^[6,7]^ Moreover, since the COVID-19 pandemic, the primary problems associated with direct, labor-intensive hands-on therapy have worsened.^[8]^ Consequently, the necessity to encourage the implementation of rehabilitation among adolescents with hemiplegic cerebral palsy (CP) throughout the COVID-19 pandemic has been enthusiastically encouraged. Reducing or eliminating the drawbacks of traditional physical therapy during CP recovery is necessary to improve the efficacy and sustainability of CP rehabilitation techniques. Current CP rehabilitation approaches, including neuromuscular conventional neurodevelopmental therapy (NDT) and proprioceptive neurodevelopmental facilitation, are widely used to address neuroplasticity and postural control in adolescents with hemiplegic CP.^[9]^

To mitigate the inherent problems of CPT, we developed an innovative deep learning-based rehabilitation application (DRA) to improve cardiopulmonary function, balance, gait, and ADL. DRA comprises inexpensive, motivational, and intensive use of smartphone camera while meeting the musculoskeletal requirements for normal gait.^[10]^ DRA platform comprises the application designed to work with a smartphone or tablet, and software algorithms virtually allow clinicians and clients free access to the platform without spatiotemporal constraints. Ample repetition is also provided through the DRA, which is designed to deliver rigorous training with little to no physical touch or stress to the therapist. Currently available deep learning games promote whole-body mobility, which enhances player engagement and movement. DRA platform is already used to detect abnormal movements, having been applied to investigate falls, measure postural sway, quantify gait performance, and assess balance ability. Generally, machine learning learns laws from a large amount of historical data through related algorithms, makes predictions or judgments on new sample data, and then learns similar to human beings. Traditional machine learning methods, such as support vector machine, hidden Markov model, random forest and other algorithms, are used to quantitatively analyze some manually specified gait features of patients to realize an automated gait analysis system.^[11]^ No experimental investigation has demonstrated how deep-learning smartphone treatments improve body mobility during rehabilitation, despite the fact that they have been demonstrated to dramatically increase patient movement compared to inactive smartphone applications. Smartphones provide thoughtfully crafted mechanisms for stimulating motivation. This study compared the effects of DRA and CPT on 6-minute walking test (6 MWT), Borg rating of perceived exertion scale (RPE), Berg balance scale (BBS), functional ambulation category (FAC), and modified Barthel index (MBI) in adolescents with hemiplegic CP. We hypothesized that there would be differences in 6 MWT, RPE, BBS, FAC, and MBI between the DRA and CPT groups.

## 2. Methods

### 2.1. Study participants

A convenience sample of 30 adolescents with hemiplegic CP (mean age = 16.8 ± 4.9 years; 4 women) was recruited from among the inpatients at the rehabilitation hospital. The experimental study protocol was approved by the local Institutional Review Board (IRB no. IRB-2023-06) and the study was performed in accordance with the tenets of Declaration of Helsinki. Informed consent was obtained from all the participants prior to their participation. The number of participants was determined to have a moderate effect size and power of 0.80 on the basis of a pilot study and power analysis.

Patients who met the following inclusion criteria were included in the study: (1) diagnosis of hemiplegic CP; (2) age ranging between 10 to 19 years; (3) ability to conduct classes with healthy children in middle and high schools; (4) gross motor function classification system (GMFCS) II–III for CP; (5) ability to directly respond to mediation and surveys; and (6) ability to follow instructions. The exclusion criteria were: (1) diagnosed with epilepsy or on antiepileptic drugs; (2) history of trauma or surgery within the last 6 months; (3) severe cognitive or visual impairments; and (4) cardiopulmonary system impairments.

### 2.2. Experimental procedure

A randomized, single-blinded, experimental design was used in the present study. Coin flipping was used to assign patients to either the control or experimental group. A researcher generated the random allocation sequence, another researcher assigned patients to interventions, and a third-party blinded researcher assessed outcome measures. To remove experimental biases associated with the patients’ expectations, experimental information that could affect the patients was masked until the experiment was completed. A procedural checklist was used to ensure consistent experimental protocols, and all instruments were calibrated daily prior to data acquisition. Standardized tests included the 6 MWT, RPE, BBS, FAC, and MBI. All tests were consistently implemented and all participants completed a demographic and health questionnaire.

### 2.3. Cardiopulmonary endurance

The 6 MWT was conducted on a level, hard-surfaced track measuring 40 m in length. In the allocated 6 minutes, each patient was free to pause and relax as needed. The length of the walk was ascertained after 6 minutes. The patient’s heart rate was continuously monitored using a portable monitor during the examination. After completing the 6 MWT, patients were asked to rate their felt level of exertion on a scale ranging from 6 (no exertion) to 20 (maximal exertion). RPE is a quantifiable measure of perceived exertion that is used to evaluate training intensity. RPE provides an indirect measure of cardiopulmonary endurance. The reliability and validity of the outcome measures have been previously established.^[12,13]^

### 2.4. Balance and gait function

Performance-oriented balance was assessed using the BBS. The examination comprised 14 basic balancing activities, ranging from standing on one foot to standing up from a sitting position. Each task was given a score ranging from 0 (unable to execute) to 4 (able to accomplish independently), and the total of sub-scores represented the degree of success. The patients’ FAC was evaluated, which is a five-point rating system that ranges from 0 (cannot walk) to 5 (can walk independently). The reliability and validity of the outcome measures have been previously established.^[13,14]^

### 2.5. ADL

The MBI is a well-established instrument for assessing a Person capacity to perform functional ADLs on their own. It is divided into 3 areas, each with a 5-point scoring system: bathing, personal hygiene, and wheelchair management; chair/bed transfers and ambulation; and feeding, dressing, using the restroom, ascending stairs, and maintaining bladder and bowel control (0–15). The range of scores was as follows: 0, complete dependence; 25 to 49, severe dependence; 50 to 74, moderate dependence; 75 to 90, mild dependence; 91 to 99, lowest dependence; and 100, independent. The reliability and validity of the outcome measures have been previously established.^[15]^

### 2.6. Intervention

**Figure 1.**
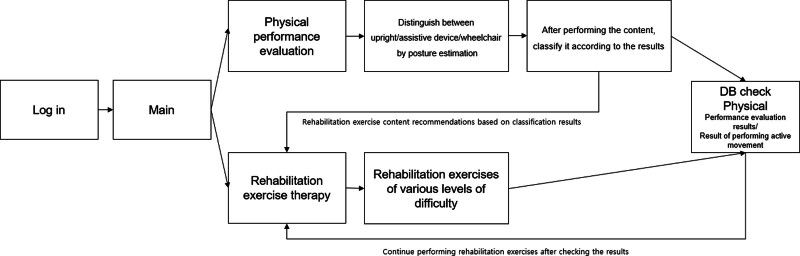
Deep learning-based rehabilitation application algorithm.

DRA and CPT were administered to the participants in a randomized manner, with each treatment session lasting 30 minutes and apportioned thrice a week for a total of 4 weeks. Examination and diagnosis revealed specific impairments. The limited activities and participation restrictions that accompanied them included general mental health issues, physical health issues that prevented one from engaging in usual role activities, emotional or physical health issues that prevented one from engaging in social activities, physical health issues that prevented one from engaging in usual role activities, and emotional health issues that prevented one from engaging in usual role activities. A licensed and experienced NDT physical therapist assisted with the study. After downloading the application, respondents completed the survey. The participants used tablets or smartphones with a stand feature. Every participant had a smart gadget that could connect to the internet at least once every 1MB/s. After downloading the application, the users selected the pertinent portion of the questionnaire. To assess whether their motions during the intervention were apparent, the participants had to allow access to the camera on their smartphones or tablets. Name, sex, birthdate, current symptoms, present since, first symptoms, and consent symptoms were included in the survey questions. Based on the vote results, the artificial intelligence (AI) determined that CP was the most likely diagnosis (Fig. 1). The researcher also noted the poll results and provided the most accurate diagnosis of CP. To ensure the participant’s safety and convenient use, a therapist and guardian are always present at the patient’s side to prevent risk of falls and safety accidents. Following the activity described in the CP referral exercise, a video of a personalized deep learning-based CP treatment intervention was played. The game was conducted with a focus on functional movement, balance training and gait training, which are mainly used in traditional physical therapy. The workout was weighted according to the user’s audiovisual feedback, and the fitness regimen was adjusted based on data from the reassessment by the deep learning convolutional neural network (CNN) system.^[15,16]^ If the participant thought that their discomfort had lessened after performing this specific action, they watched the movie again.

**Figure 2.**
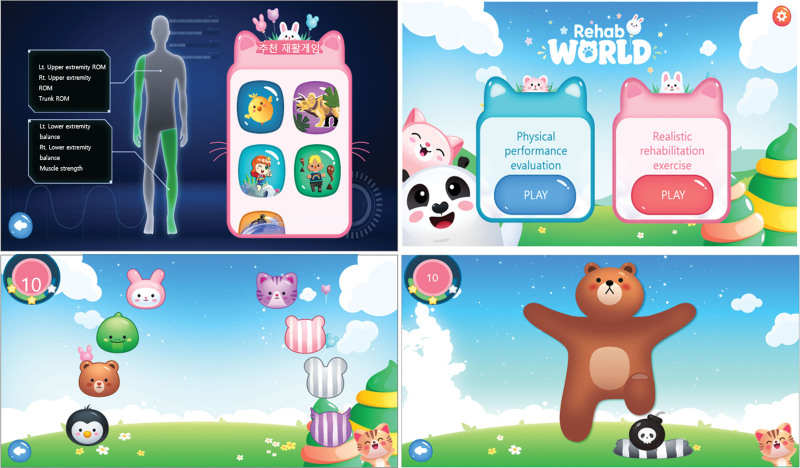
Deep learning-based rehabilitation application platform.

The deep learning CNN system was used to modify the fitness routine based on the information obtained from reassessment and weighing the workout depending on the user’s audiovisual feedback.^[16]^ The CNN served as the foundation for deep learning motion analysis method used in this study.^[17]^ The most frequently used AI algorithm in clinical settings can handle erroneous data, carry out nonlinear data analysis, and pick up knowledge from prior instances. The program used neurons to read data from subjective evaluation (input layer) and chose the most beneficial therapeutic workouts from the exercise library (hidden layer).^[18]^ Next, the output layer demonstrated the ideal therapy program. Backpropagation training was used after CNN had completed its first weighting training using the given samples and continued until at least 80% accuracy was attained. By contrasting the workout plan produced by deep learning with one designed by experts, the model was validated.^[19]^ A CNN-based smartphone camera is one potential application for DRA in real-time and on-site monitoring for CP movement (Fig. 2).

Task-oriented movement training and recent NDT research are the sources of inspiration for the manual treatment approach included in the CPT.^[20]^ The neurodevelopment sequence comprised the following exercises: supine-side lying-prone rolling, sitting, kneeling and half-kneeling, transfer, plantigrade, standing, and walking training. The 25-min NDT exercises included core stabilization, motor control stages (mobility, stability, controlled mobility, and skill), and inhibitory and facilitatory motor control. Since each patient received care from the same licensed therapist in accordance with the most recent system requirements, the NDT-Bobath concept or method was guaranteed.^[21]^ A component of the intervention is teaching functional skills including sensory, perceptual, and adaptive components. Sensorimotor experience must be incorporated into activities as learning is based on movement perception. Enhancing gross motor function in children and individuals with neurological disorders is the aim of neurodevelopmental therapy, which aims to improve their independence in several contexts.^[22]^ It is contemplated that by stimulating the affected side to promote appropriate muscle activation, abnormal movement patterns that are appropriate for performing daily tasks can be healed and normal movement patterns restored. CPT includes facilitation (using sensory inputs to improve motor performance), control of compensatory motor activity, and a comprehensive management plan. It is believed that aberrant movement patterns can be repaired and normal movement patterns favorable to carrying out daily activities can be restored by stimulating the afflicted side to encourage the desired muscle activation. Three main components of CPT include control of compensatory motor behavior, facilitation (using sensory inputs to increase motor performance), and an overall management approach. According to Kollen et al (2009), the patient has to participate actively in receiving assistance from the therapist. Using important areas of control including the head, shoulders, and pelvis, the therapist leads the patient’s entire body movement while assisting with their movement.^[23]^ Task-specific stances and motions are used in CPT. It places a strong emphasis on engaging in relevant everyday circumstances and functional tasks. CPT’s primary goal is to maximize patients’ activity and involvement levels in order to enhance their quality of life despite neurological impairments. The development of the intervention is modified according to the functional level of each patient.

### 2.7. Statistical analysis

Results were presented as means and standard deviations. All variables were analyzed using the Kolmogorov–Smirnov test, assuming a normal distribution. A power analysis, applying G-Power software, was performed to determine the sample size requirement (N = 27) based on our pilot study, which showed effect size (eta squared, η^2^ = 0.5) and power (1-β = 0.8). Considering a dropout rate of 10%, 30 participants were recruited. Chi-square tests were used to determine variability across sexes. Independent *t*-tests were used to define demographic data and clinical outcome measurements. A two-group, two-intervention, repeated-measures analysis of variance (ANOVA) was used to identify the 6 MWT, RPE, BBS, FAC, and MBI. Tukey post hoc analysis was used to assess whether the interaction and main effects were examined. The time effect refers to the difference between before and after intervention. SPSS software (version 26.0, SPSS, Chicago, IL) was used to perform all statistical analyses. The *P*-value was set to .05.

## 3. Results

### 3.1. Demographic characteristics of the study participants

The demographic characteristics of the participants are presented in Table 1. All the participants successfully completed the experimental tests and interventions. There were no significant differences between the DRA and CPT groups in terms of sex, age, height, weight, or side of hemiplegia (*P* > .05).

**Table 1:** Demographic characteristics of the study participants (N = 30).

Characteristics	DRA (n = 15)	CPT (n = 15)	*P*-value
Age (years) Height (cm) Weight (kg)	15.47 ± 3.67 143.43 ± 9.48 40.15 ± 5.37	13.19 ± 2.8 145.96 ± 7.84 35.02 ± 10.37	.708 .28 .069
Sex			
Male (%)	13 (87)	13 (87)	1.000
Female (%)	2 (13)	2 (13)	
Side of hemiplegia			
Left (%)	10 (66)	9 (60)	.977
Right (%)	5 (34)	6 (40)	
Type of cerebral palsy			
Spastic diplegia	15 (100)	15 (100)	1.000
MAS			
1	10 (66)	9 (60)	.977
1+	5 (34)	6 (40)	
WISC V	100.18 ± 10.66	98.79 ± 14.28	.224

CPT = conventional physical therapy; DRA = deep learning-based rehabilitation application; MAS = modified Ashworth scale; WISC V = Wechsler intelligence scale for children.

### 3.2. Cardiopulmonary function

Repeated-measures ANOVA showed significant effects of both DRA and CPT interventions on the 6 MWT (*P* = .001) and BRE (*P* = .001). Moreover, a significant difference in the 6 MWT distance was observed between the two groups (*P* = .031). Paired *t*-test showed significant differences in the 6 MWT distance between the DRA pre- and posttests. Post hoc analysis showed that DRA resulted in a greater increase in the 6 MWT distance than CPT (Table 2).

**Table 2:** Cardiopulmonary function.

	DRA		CPT		*P*-value		
	Pretest	Posttest	Pretest	Posttest	Time effect	Between groups	Time × group
6 MWT	531.11 ± 28.81	581.13 ± 17.13	493.18 ± 13.88	527.83 ± 24.13	0.001*	0.03*	0.001*
BRE	12.13 ± 1.88	13.11 ± 0.83	11.84 ± 2.13	13.01 ± 0.77	0.001*	0.27	0.18

6 MWT = 6-minute walking test; BRE: Borg rating of perceived exertion scale; CPT = conventional physical therapy; DRA = deep learning-based rehabilitation application.

* *P* < 0.05.

### 3.3. Clinical outcome and gait comparison

Repeated-measures ANOVA showed significant effects of both DRA and CPT interventions on the BBS (*P* = .001) and MBI (*P* = .001). Moreover, a significant difference in BBS score and MBI was observed between the two groups (*P* = .03; .006). Paired *t*-test showed significant differences in the BBS score and MBI between the DRA pre- and posttests. post hoc analysis showed that DRA resulted in a greater increase in BBS and MBI than CPT (Table 3).

**Table 3:** Clinical outcome and gait comparison.

	DRA		CPT		*P*-value		
	Pretest	Post-test	Pretest	Post-test	Time effect	Between groups	Time × group
BBS	24.84 ± 9.31	36.11 ± 8.14	22.64 ± 10.11	31.83 ± 8.79	0.001*	0.03*	0.001*
FAC	3.81 ± 0.69	3.93 ± 0.76	3.71 ± 0.25	3.88 ± 0.12	0.08	0.11	0.14
MBI	38.11 ± 16.46	48.03 ± 17.12	34.55 ± 28.14	40.81 ± 18.88	0.001*	0.006*	0.003*

BBS = Berg balance scale; CPT = conventional physical therapy; DRA = deep learning-based rehabilitation application; FAC = functional ambulation category; MBI = modified Barthel index.

* *P* < 0.05.

## 4. Discussion

To the best of our knowledge, this study provides the first novel evidence highlighting the clinical effects of deep learning-based rehabilitation on cardiopulmonary function, dynamic and static balance, gait function, and ADL in adolescents with hemiplegic CP. As hypothesized, the DRA group showed significant differences compared to the CPT group in terms of cardiopulmonary function, dynamic and static balance, gait function, and ADL. Importantly, the DRA group demonstrated clinically more meaningful improvements in 6 MWT, BBS, and MBI in comparison to the CPT group.

Cardiopulmonary function analysis demonstrated that the improvement in 6 MWT (2.39%) was significantly different between the DRA and CPT groups. This finding was consistent with the results of Rooji et al (2021), who reported an increase in 6 MWT (36.5%) among patients with hemiplegic stroke after 6 weeks of virtual reality rehabilitation program in comparison to the control group.^[24]^

Park et al (2017) reported an increase in 6 MWT (27.1%) following 6 weeks of Kinect gaming compared to conventional rehabilitation program in 20 patients with hemiplegic stroke.^[25]^ Lesmana et al (2023) demonstrated an increase in 6 MWT (4.03%), heart rate (22.34%), and oxygen saturation (1.27%) after 24 sessions of virtual reality cycling exercise compared to pre-intervention in 8 patients with hemiplegic stroke.^[26]^ The results of aforementioned studies indicated the use of different virtual platform environments; however, all interventions were intended to provide functional, personalized, and progressive training. In addition, there are other explanations that do not provide the greatest opportunity to customize the virtual platform. There is some evidence that virtual platforms focus on safety and maximization aspects of walking function.^[27]^ In the context of enhancement of cardiopulmonary function using deep-learning-based interventions, such as stationary walking, squatting, or upper/lower extremity exercise, improved cardiopulmonary function outcomes were obtained. This could be due to the influence of task-specific or goal-directed training in which patients with hemiplegic stroke practice context-specific motor tasks and receive feedback. Thus, task specificity seems to be a crucial but not the only factor in the use of deep learning-based interventions.^[28]^ In addition, the intensity of the intervention could be considered a dosage and timed exercise. In this study, the intensity of DRA intervention surmounted to 30 minutes per session for a total duration of 12 sessions. Neuroscience studies have shown that long-term, high-intensity rehabilitation training regimens can enhance motor capabilities by reorganizing neural networks and causing neuronal plasticity.^[28–30]^ A scoping review also discovered, in contrast to another one, that many studies included psychological outcomes, including immediate intrinsic motivation, anxiety, depression, and mood.^[28,31–33]^

ADL and balance analysis indicated that improvement in BBS (4.78%) was significantly different between the DRA and CPT groups. This finding was in concordance with a previous study by Sana et al (2023) who reported increased improvement in balance (11.1%) following 8 weeks of virtual reality therapy than vestibular rehabilitation therapy in 34 patients with subacute hemiplegic stroke.^[34]^ Pelaez-velez et al (2023) also demonstrated increased improvement in balance (18.58%) among patients with hemiplegic stroke following 6 weeks of virtual reality therapy in comparison to the control group.^[35]^ Lee et al (2023) indicated increased improvement in ADL (8.32%) in 4 patients with hemiplegic stroke.^[36]^ By applying audiovisual feedback on deep learning-based rehabilitation, the patients are able to self-train balance deficits by analyzing the postural and balance control suggestions shown in the virtual environment. DRA is a low-cost, portable tool that can be integrated into the rehabilitation plan of post-CP and neurological patients, such as those with traumatic brain injury and stroke. It remains difficult to produce individualized software with exercises tailored to the patient’s demands and improve movement detection systems. Cooperation between the entertainment and medical sectors may offer intriguing solutions for creating more accessible and realistic virtual workout regimens. The ability to support and include neurocognitive and neurorehabilitation techniques, such as action recognition, motor imaging, and movement guidance, is a benefit of DRAs. All these methods are based on neurophysiological evidence that the motor system is involved in movement in ways other than just executing motor actions, such as recognizing dynamic movements, processing verbal action information, and motor imagery.^[33,34]^ The motor system as a whole is activated anytime an action is performed, visualized, identified, or spoken. Action reenactment is the ability to use the same brain areas involved in the execution of actions for cognitive processes, such as action recognition and motor imagery. Additionally, as demonstrated in previous research, DRA has favorable medium-term benefits in reducing depression symptoms, boosting motivation, and enhancing neurorehabilitation variability. Motivated patients play an active role in their rehabilitation and are more focused on finishing the assigned task.^[37]^ Additionally, the combination of peripheral nerve activation and tactile sensations may facilitate the reorganization of cerebral connections, which would enhance motor capability. Such rehabilitation frequently requires sophisticated and costly equipment that is only available in hospitals or other specialized facilities.^[36,37]^ DRA not only provides easy and affordable virtual treatment options, but also exhibits positive outcomes in the ongoing recovery of chronic CP survivors.

This study had two limitations that should be addressed in future research. First, despite our extensive measurements of clinical balance, ADL, and cardiopulmonary condition, we were unable to measure the mechanism underlying the change in neuroplasticity or the results of exercise stress test because quantitative measuring tools were not available. Second, the lack of follow-up evaluation and satisfaction survey, which can have important effects on the sustainable therapeutic effects of DRA in adolescents with hemiplegic CP.

## 5. Conclusion

Clinical research has demonstrated that deep learning-facilitated rehabilitation is beneficial for balance, ADL, and cardiopulmonary function in adolescents with hemiplegic CP. Our novel results provide clinical evidence that DRA improves the recovery of cardiopulmonary function, dynamic and static balance, and ADLs such as bathing, clothing, and dressing. Most importantly, the DRA platform allows autonomous liberty to provide accurate real-time quantitative audiovisual feedback and effective rehabilitation training, which could serve as a basis for advanced robotic science and medical research.

## Author contributions

**Conceptualization:** Yeongsang An, Chanhee Park.

**Data curation:** Yeongsang An, Chanhee Park.

**Formal analysis:** Yeongsang An, Chanhee Park.

**Investigation:** Yeongsang An, Chanhee Park.

**Methodology:** Yeongsang An, Chanhee Park.

**Project administration:** Chanhee Park.

**Software:** Seunghwa Min, Chanhee Park.

**Supervision:** Seunghwa Min, Chanhee Park.

**Validation:** Seunghwa Min.

**Visualization:** Seunghwa Min.

**Writing – original draft:** Seunghwa Min, Chanhee Park.

**Writing – review & editing:** Seunghwa Min, Chanhee Park.
